# How do SNP ascertainment schemes and population demographics affect inferences about population history?

**DOI:** 10.1186/s12864-015-1469-5

**Published:** 2015-04-03

**Authors:** Emily Jane McTavish, David M Hillis

**Affiliations:** Department of Ecology and Evolutionary Biology, University of Kansas, 1200 Sunnyside Avenue, Lawrence, KS 66045 USA; Heidelberg Institute for Theoretical Studies, Schloss-Wolfsbrunnenweg 35, Heidelberg, D-69118 Germany; Department of Integrative Biology, University of Texas, One University Station C0990, Austin, TX 78712 USA

**Keywords:** *Bos taurus*, *Bos indicus*, Gene-flow, Migration, SNP chip

## Abstract

**Background:**

The selection of variable sites for inclusion in genomic analyses can influence results, especially when exemplar populations are used to determine polymorphic sites. We tested the impact of ascertainment bias on the inference of population genetic parameters using empirical and simulated data representing the three major continental groups of cattle: European, African, and Indian. We simulated data under three demographic models. Each simulated data set was subjected to three ascertainment schemes: (I) random selection; (II) geographically biased selection; and (III) selection biased toward loci polymorphic in multiple groups. Empirical data comprised samples of 25 individuals representing each continental group. These cattle were genotyped for 47,506 loci from the bovine 50 K SNP panel. We compared the inference of population histories for the empirical and simulated data sets across different ascertainment conditions using *F*_*ST*_ and principal components analysis (PCA).

**Results:**

Bias toward shared polymorphism across continental groups is apparent in the empirical SNP data. Bias toward uneven levels of within-group polymorphism decreases estimates of *F*_*ST*_ between groups. Subpopulation-biased selection of SNPs changes the weighting of principal component axes and can affect inferences about proportions of admixture and population histories using PCA. PCA-based inferences of population relationships are largely congruent across types of ascertainment bias, even when ascertainment bias is strong.

**Conclusions:**

Analyses of ascertainment bias in genomic data have largely been conducted on human data. As genomic analyses are being applied to non-model organisms, and across taxa with deeper divergences, care must be taken to consider the potential for bias in ascertainment of variation to affect inferences. Estimates of *F*_*ST*_, time of separation, and population divergence as estimated by principal components analysis can be misleading if this bias is not taken into account.

**Electronic supplementary material:**

The online version of this article (doi:10.1186/s12864-015-1469-5) contains supplementary material, which is available to authorized users.

## Background

Next-generation sequencing has made genomic sequence data available even in many non-model organisms. Broader analysis of genetic variation across many individuals or populations within species typically relies on methods that subsample variable sites within genomes. One of the most efficient and widely used approaches for comparing genomic variation within species uses single nucleotide polymorphism (SNP) panels [1,2]. SNP panel methods rely on deeply sequencing a subset of the population of interest and then using this information to select polymorphic loci for additional genotyping in a much larger pool of individuals, often using chip-based genotyping. However, a bias present in the initial selection of markers may affect inferences about the larger population. In this study, we investigated the effects of this selection bias on inferences of demographic history using an empirical example from cattle.

Standardizing SNP panels, as was done for the Human Hap-Map project [3], makes it straightforward for research groups to combine data and address a broad array of biological questions. For example, SNP-panel analyses have been used extensively for disease research (reviewed in [4]). Commercial direct-to-consumer applications of SNP-panel genotyping allow individuals to trace their ancestry and test for disease-associated SNPs [5]. Novembre *et al.* [6] used SNP loci genotyped for the POPRES project [7] to analyze the genetic spatial structure of human populations in Europe. Chip-based SNP sequencing is also available for several plants and animals of scientific or agricultural importance, including dogs, mice, cattle, chickens, horses, pigs, sheep, and corn [http://www.neogen.com/geneseek/SNP_Illumina.html]. Chip-based SNP analyses have been used to resolve evolutionary relationships in extinct ruminants [8], and to understand global patterns of population structure in cattle and dogs [9-11]. SNP sets are also being developed for conservation applications [12] and have been used to test for hybridization between common and endangered species (e.g. [13-15]).

To discover variable SNP loci for inclusion in a SNP panel, a sample of individuals representing the taxon of interest is sequenced. This sample of individuals is called the “ascertainment group.” The ascertainment group’s size and composition is determined by the developers of the panel, and typically depends on the aims of the study at hand. A set of SNPs is then selected from the resequencing data of the ascertainment group. The selection of individuals used for the ascertainment group can bias which SNPs are discovered and included in later genotyping analyses.

Ascertainment bias is of course not unique to SNP analyses. For example, in morphological analyses, variable traits are often preferentially selected over fixed traits for analysis. Furthermore, in microsatellite or gene sequencing studies, genes are often chosen for sequencing based on their levels of variability within a group of interest [16]. Arnold *et al.* [17] recently demonstrated that RAD sequencing introduces genealogical biases due to nonrandom haplotype sampling. All of these forms of ascertainment bias influence the variability of the sampled data relative to the expectations for data sampled at random from the genome.

There are two main forms of ascertainment bias associated with SNP-panel analyses: minor allele frequency (MAF) bias and subpopulation bias. MAF bias results in the over-representation of polymorphisms with high minor allele frequencies and the under-representation of polymorphisms with low minor allele frequencies. The number of individuals in the ascertainment group will influence the lower frequency limits of SNPs included on the SNP panel. Mutations that are less common than 1/*n*, where *n* is the number of alleles in the panel, are unlikely to be observed in the ascertainment group. Much research has been devoted to describing and mitigating the impacts of minor allele frequency cut-offs in the generation of SNP panels [18-21].

In this study we addressed the issue of subpopulation bias in ascertainment. This bias arises from the selection of individuals to include in an ascertainment panel. If the panel is chosen from individuals from a subpopulation or geographic region, variability in that group will be over-represented [22,23]. Wang and Nielsen [24] addressed phylogenetic aspects of ascertainment bias in an outgroup of the taxon of interest. Excoffier *et al.* [25] developed a simulation-based framework, *fastsimcoal2*, which can accurately infer demographic parameters for even very complex models under known ascertainment schemes (such as markers heterozygous in a single individual). Subpopulation bias in the composition of the group used to select variable markers can also affect inferences using those markers. For example, microsatellite repeat loci are consistently longer in the species in which they are discovered than in other species in which they are amplified [26]. Subpopulation ascertainment can inflate heterozygosity and apparent diversity in populations closely related to the ascertainment group [20,21,27-30]. Using simulated and empirical data for 30 restriction-site polymorphism markers, Eller [30] demonstrated that ascertainment-group bias can artificially inflate within-group estimates of diversity, especially when real heterozygosity is low. The effects of subpopulation bias in genomic data needs further exploration, particularly as it affects studies of non-humans. The bulk of these analyses of SNP ascertainment bias have been performed on human data [20,24,25,27-31], where among population divergences are necessarily limited. As genomic analyses are expanding into analyses of non-model organisms, it is essential to investigate these issues across broader time-scales and in other organisms.

This study examines on the impact of subpopulation ascertainment bias on population demographic inference using *F*_*ST*_ values and principal components analysis (PCA). *F*_*ST*_ is a frequently used measure of population differentiation that summarizes differentiation between groups [32]. PCA is a statistical method for reducing the dimensionality of data that can be used for inferring population structure from genetic data (e.g. [33,34]). The first two principal component (PC) axes of human SNP data are correlated strongly with spatial coordinates [6]. PCA has been widely applied to inferring spatial genetic structure using SNP data in humans (e.g., [35,36]; as well as other species (e.g., cattle: [10]; and dogs: [11]). McVean [37] described a genealogical interpretation of the principal component axes for SNP data, where the first PC axis is expected to capture the deepest coalescent split in a tree. In addition, relative PC components can be used to infer admixture between ancestral populations [37].

### Study system

To test the effects of subpopulation-biased ascertainment on inference of population histories, we simulated data based on demographic models of cattle evolution [38,39]. Domesticated cattle are comprised of lineages derived from two independent domestication events: the taurine and indicine lineages. Indicine cattle are common in the Indian subcontinent and taurine cattle are common in Europe; an African taurine lineage as well as indicine cattle and hybrid lineages exist in Africa. Taurine and indicine cattle likely share a most recent common ancestor 200,000 or more years ago (84*–*219 thousand years ago [kya]: [40]; 260*–*300 kya: [38]; 335 kya: [41]; 200 kya*–*1 mya: [42]). The divergence between African and European taurine cattle is much more recent (9*–*15 kya: [40]; 10*–*15 kya: [41]; 12.5 kya: [43]). This divergence represents the major population structuring within taurine cattle. In addition, there is a several-thousand-year history of admixture between taurine and indicine lineages in Africa [44]. This range is consistent with either a single domestication of taurine cattle, or an independent African domestication event.

We compared data simulated under three demographic models to empirical data for samples of European, African and Indian cattle collected using a 50 K-marker bovine SNP chip [45]. The 50K SNP panel was generated by a complex ascertainment scheme including taurine, indicine, and hybrid African breeds, but it is biased toward capturing polymorphisms that segregate in European breeds, as well as polymorphisms that are shared between taurine and indicine cattle [45]. It under-represents sites that are fixed differences between taurine and indicine lineages, or are polymorphic only in indicine cattle [45]. The minor allele frequency cut off was an average marker (MAF) of at least 0.15 among common cattle breeds, including both taurine and indicine cattle [45].

Cattle are a useful system to investigate the effects of ascertainment bias because there exist well-parameterized demographic models based on sequence data that allow us to simulate large unbiased data sets. In addition, domesticated cattle comprise groups (the taurine and indicine lineages) with deep divergences between them. Therefore, cattle represent a good system to explore the effects of capturing SNP loci across subspecies or species boundaries.

## Methods

The term “SNP” is commonly used to mean “variable site” across samples irrespective of whether a given SNP is polymorphic within a population. Although Wakeley *et al.* [46] coined the more accurate term “SNP-discovered locus” (SDL) to describe these single nucleotide differences that may or may not be segregating within sampled groups, this terminology is not widely used. Here, we use SNP in the broad sense of “variable site.”

### Empirical data

Our empirical data set consisted of a subset of the cattle SNP data described in McTavish *et al.* [10]. We used genotypes for 25 individuals from each of three breeds representative of the three major geographic clusters of cattle: Indian (Gir), African (N’Dama), and European (Shorthorn). The African (N’Dama) samples are from a group with largely African taurine ancestry, but have some indicine introgression [10]. We included all 25 Gir samples from the published data set. The 25 Shorthorn individuals included were a random subset of the total set of Shorthorn samples (n = 99). The 25 N’Dama individuals included were a random subset of the N’Dama samples excluding 13 individuals estimated to have admixed ancestry within the last 100 years ([47]; n = 46). The loci examined consisted of 47,506 SNPs genotyped using the bovine 50 K SNP chip [45]. This subset of markers was selected by removing loci that had >10% missing data across a larger sample of 1,420 cattle [10]. There were no ambiguous or absent base calls in the analyzed SNP data matrix, as the larger data set had been filtered and missing data imputed as described in McTavish *et al.* [10].

### Demographic model

**Figure 1:**
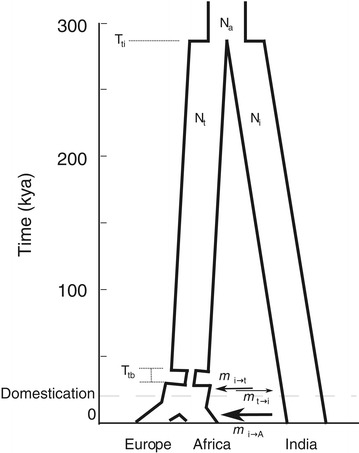
**Demographic model used for simulations.** Parameter values are described in Table 1. Arrows represent migration between populations. Arrow width is representative of relative values of these migration parameters under demographic scenario *c*. Figure created using *MatPlotLib* [76] in *IPython* [77].

We simulated data under a demographic model for population structure in domesticated cattle and their wild ancestor, the aurochs (Figure 1, Table 1). In this model taurine and indicine lineages share a most recent common ancestor 280,000 years ago (T_ti_) [38,42]. The ancestral population size (N_a_) is 15,000 individuals (rounded from 14,127 in [38]). A bottleneck reducing the population size to 150 individuals (0.01*N_*a*_) occurred in the taurine lineage from 40–36 kya (T_tb_), followed by a population expansion to 19,212 (1.36*N_a_; parameters from [38]). In contrast, indicine lineage population remained constant [39]. Within the taurine lineage, the divergence between European and African cattle occurred 15,000 years before present. This value is at the older end of a spectrum of divergence time estimates for European and African taurine cattle (9*–*15 kya: [40]; 10*–*15 kya: [41]; 12.5 kya: [43]). We assumed a generation time of 5 years for both aurochs and domesticated cattle [38,48].

**Table 1:** **Parameter values for the three demographic models simulated, shown in Figure** 1

**Variable**	**Description**	***a***	***b***	***c***
	Generation time	5 years	-	-
N_a_ = N_t_ = N_i_	Ancestral population sizes	15,000	-	-
N_tE_	Current European taurine population size	7,500	-	-
N_tA_	Current African taurine population size	7,500	-	-
N_i_	Current indicine population size	15,000	-	-
T_AE_	Time of African–European divergence	15 kya (3,000 generations)	-	-
T_tb_	Timing of bottleneck in taurine cattle	40–36 kya	-	-
N_tb_	Size of bottleneck in taurine cattle	150 (0.01 × N_a_)	-	-
T_ti_	Time of indicine–taurine divergence	280 kya (56,000 generations)	-	-
*m*_i→t_	Number of migrants from indicine to taurine lineages per generation (prior to European–African split 15 kya) (Murray *et al.* 2010 [38])	0	0.2175	0.2175
*m*_t→i_	Number of migrants from taurine to indicine lineages per generation (prior to European–African split 15 kya) (Murray *et al.* 2010 [38])	0	0.0125	0.0125
*m*_i→A_	Number of migrants from indicine lineages into Africa per generation for the past 15 kya	0	0	2

Parameter values adapted from Murray *et al.* [38].

Values for simulations (*b*) and (*c*) were the same as for (*a*) unless specified.

We simulated data with this demographic model under three different migration conditions (full parameters in Table 1, Additional file 1: Table S1): (*a*) no migration; (*b*) low levels of asymmetric gene flow (migration) as estimated from nuclear sequence data in [38] between indicine and taurine lineages equivalent to indicine to taurine gene flow of 1 migrant every 4.6 generations (*m*_i→t_), and lower taurine to indicine gene flow of 1 migrant every 80 generations (*m*_t→i_); and (*c*) migration as described in *b* plus moderate levels of gene flow equivalent to 2 individuals per generation from indicine lineages into the African taurine population from 15 kya to present (*m*_i→A_).

### Simulation software

We simulated demographic histories using the software *ms* [49]. The *ms* program is a backwards-in-time coalescent simulator that generates samples according to a Wright–Fisher neutral model. We used *ms* to generate both gene trees and samples of variable sites for each migration scenario. To match our simulated data to the empirically generated data set, we simulated samples of 50 haplotypes at 47,506 variable loci for each of the groups of European, Indian, and African cattle. We paired consecutive haplotypes to create diploid genotypes. The software *ms* uses θ (4*N*_*0*_*μ*) where *N*_*0*_ is the diploid population size, and *μ* is the neutral mutation rate for the locus. As we were interested only in variable sites, we used a high neutral mutation rate (3x10^-6^) and included only sites at which a mutation had occurred. All markers were variable with respect to the 150 simulated haplotypes. We did not use a within-group minor allele frequency cutoff. Each simulated locus was independent and unlinked from all others. The infinite sites assumption of the *ms* model prevents multiple mutations at the same site from occurring. The commands we used are listed in the supplemental information (Additional file 1: Table S1). We replicated the simulations five times.

### Ascertainment schemes

We subjected each of these simulated migration conditions to three SNP ascertainment treatments. We selected 1,000 SNPs under each of the following ascertainment schemes: (I) *Random*: SNPs were selected at random without replacement; (II) *Geographically-biased:* 800 SNPs were selected from loci that were polymorphic in Europe, regardless of polymorphism in other groups, and 200 SNPs were selected randomly; and (III) *Polymorphism-biased:* 800 SNPs were selected from SNPs that were polymorphic in more than one group. Under this polymorphism biased scheme SNPs that were polymorphic in all three groups were four times as likely to be selected as those only polymorphic in two groups. 200 SNPs were selected randomly.

The simulation process generated five 47,506-SNP replicates for each of the three demographic scenarios (*a, b,* and *c*). For each of the simulated data sets we created 1,000-marker subsamples under each of our three ascertainment schemes (I, II, and III). For the observed data set we created five 1,000-marker random subsamples. This replication allows us to test for statistical significance of results, and to compare variation among samples of the observed data to that within and between the simulated samples. We performed the analyses described below on each of five replicates for the nine migration by ascertainment scheme conditions ([*a*, *b*, *c*] * [I, II, III]), and compared the parameter values and variances to those calculated from five 1,000-SNP random subsamples of the empirical data set.

### Population genetic parameters

We calculated the number of polymorphic sites in each continental group (European, African, Indian) in each of the empirical and simulated data sets. We calculated pairwise *F*_*ST*_ for all pairs of populations for the subsampled data using Weir and Cockerham’s [50] method implemented in *Genepop* 4.2 [51]. We calculated the mean and standard deviation of the *F*_*ST*_ values across the five simulation runs. We tested for differences among and interactions between demographic scenarios and ascertainment schemes for pairwise *F*_*ST*_ values using two way analysis of variance (ANOVA) using the StatsModels package in Python [52].

### Principal components analysis

We performed principal components analysis on each sampled data set using *smartpca* in the EIGENSTRAT software package [53]. We calculated the average proportion of variation explained by PC1 and PC2 under each condition across the five simulation runs. Analysis of variance (ANOVA) on these values was performed with the *stats.f_oneway* function in SciPy [54]. Additional PC axes captured within-population variation and were not further explored. We compared the major axes of variation in the PCA and the proportion of variation explained by each PC axis between data sets generated under each of these ascertainment schemes [54].

### Goodness-of-fit tests

To test the goodness of fit of alternative demographic models to our observed data, we calculated the percentage of polymorphisms falling into each of seven categories: (1) segregating only in the European lineage; (2) segregating only in the African lineage; (3) segregating only in the Indian lineage; (4) segregating in the European and African lineages; (5) segregating in the Indian and European lineages; (6) segregating in the Indian and African lineages; and (7) segregating among all three lineages. In each of our five replicate runs we calculated the absolute difference between the empirical percentages observed in each category and the percentages observed in simulated replicates. We summed these percentages to create a quantitative measure of the degree of match. The lower the sum of absolute differences, the closer the fit. We did not perform significance tests on these deviations as we had no null expectations for their values.

To measure goodness of fit for the simulated principal components analyses, we took two approaches. First, we calculated the estimated admixture proportions of the African cattle. Admixture between two population groups for an individual may be estimated using PCA by calculating the relative position along the major PC axis differentiating those groups [37]. Second, we used Procrustes analysis to compare the spatial relationships of PC coordinates across different migration and ascertainment schemes [55,56]. Procrustes analysis applies rotation and scaling to coordinates to minimize the Euclidean distance among individuals across analyses. This provides a metric of differences in the spatial orientation of observed points in two dimensions, and thus allows us to compare patterns across the entire PCA results between analyses. We used the Procrustes function in the R package *vegan* to perform Procrustes superposition and calculate the residual sums of squares, and performed a test of significance of similarity of coordinates using *PROTEST* [57,58]. These values were calculated for comparisons of the simulated data sets to the observed data across the five 1,000 SNP replicates.

## Results

**Figure 2:**
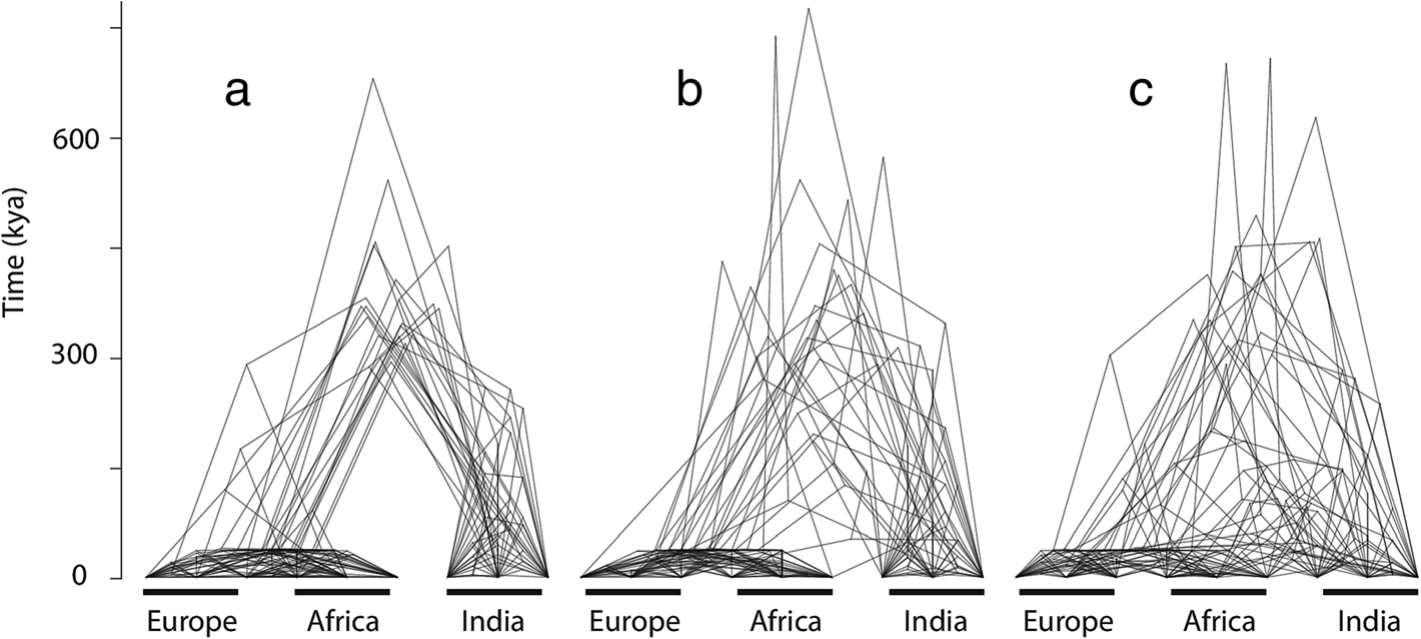
**Gene trees generated according to the demographic models under each of three migration scenarios.** Gene trees are plotted atop one another so that patterns of variation among loci are visible. **(** ***a*** **)** No migration; **(** ***b*** **)** low taurine–indicine gene flow; and **(** ***c*** **)** low taurine–indicine gene flow, plus higher recent indicine to Africa gene flow. Figure created using the *Densitree* function [78] in the *phangorn* package [79] of R [57].

We generated five replicates of 47,506 polymorphic loci for 150 sampled haplotypes under three migration scenarios: (*a*) no migration; (*b*) low asymmetric taurine–indicine gene flow since domestication; and (*c*) low asymmetric taurine–indicine gene flow since domestication, combined with higher recent indicine to Africa gene flow. We also sampled 30 simulated gene trees under each of these demographic scenarios (Figure 2).

### Distribution of polymorphisms

**Figure 3:**
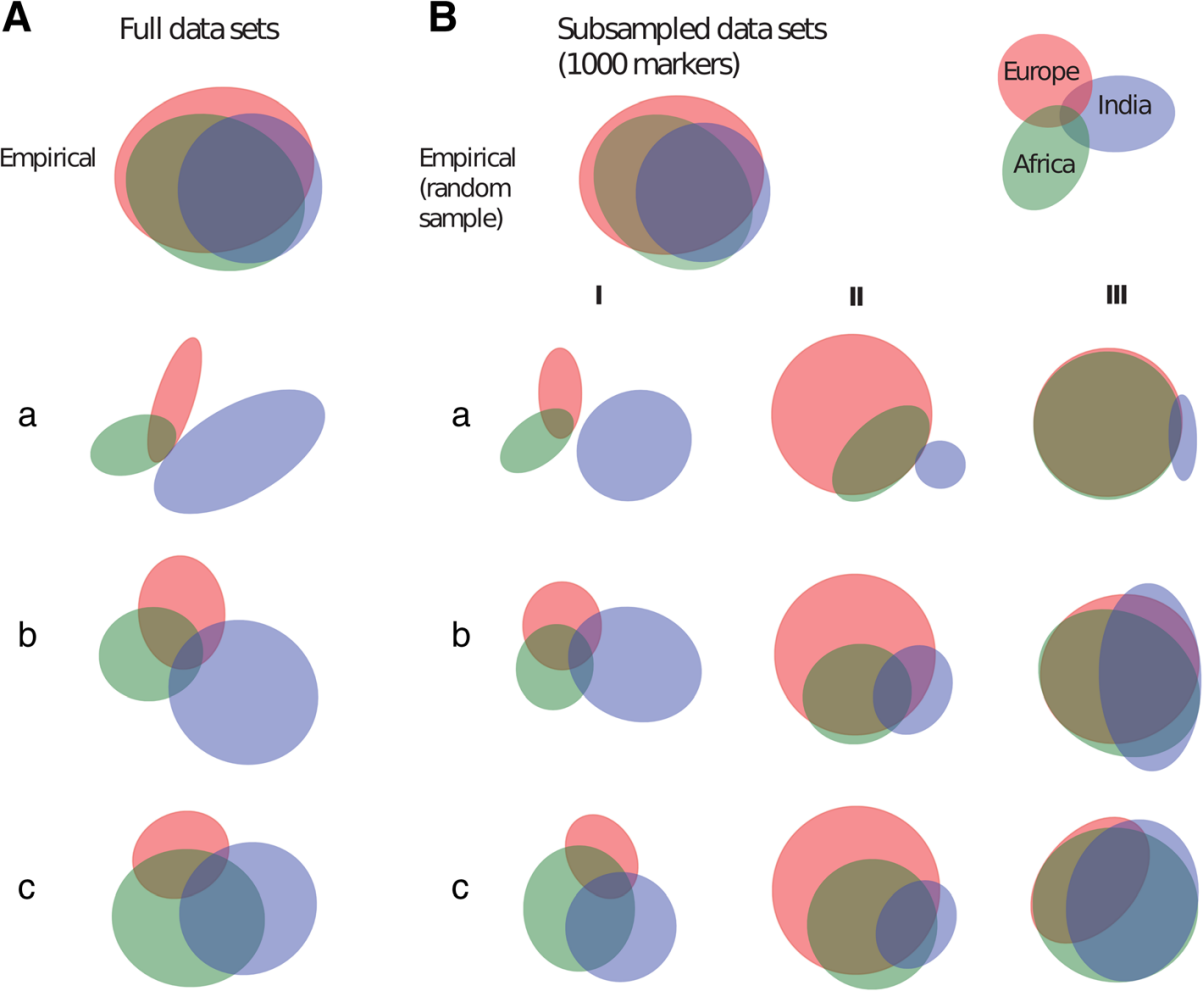
**Venn diagrams illustrate the counts of polymorphisms segregating within each continental group for one example replicate.** Sizes of circles and areas of overlap are approximately proportional to number of sites in those categories. Fixed differences between populations are not shown here. **(A)** Full data sets for the empirical data and the three simulated data sets. **(B)** 1,000-marker subsets of the empirical data set and the simulated data sets. Three demographic conditions were analyzed: (*a*) No migration; (*b*) low taurine–indicine gene flow; and (*c*) low taurine–indicine gene flow, plus higher recent indicine to Africa gene flow. In addition, three types of ascertainment sampling scheme were applied: (I) SNPs were based on random samples of loci (no bias); (II) sampled loci were selected from those that were polymorphic within Europe; and (III) sampled loci were selected from loci that were polymorphic in two or more subpopulations. Figure made using *EulerAPE* [80]. Counts of polymorphisms in all groups are shown in Additional file 1: Table S2.

The distributions of polymorphisms across groups were very different among simulated and empirical data sets, and are compared in Figure 3 and reported in Additional file 1: Table S2. This figure and accompanying table represent only a single demographic simulation replicate for ease of visualization. Additional file 1: Table S3 reflects the deviations across all replicates. Although all sites were polymorphic with respect to the full sample of 75 diploid individuals, many represented fixed differences between populations that were not polymorphic within any of the three subgroups. The number of sites that were polymorphic within at least one population varied among the three demographic scenarios as follows: (*a*) no-migration demographic scenario: 27,822 sites; (*b*) low taurine–indicine gene flow demographic scenario: 32,611 sites; and (*c*) low taurine–indicine gene flow plus higher recent indicine to Africa gene flow demographic scenario: 36,635 sites. The lowest absolute deviation between observed and simulated polymorphism counts was under moderate migration (demographic scenario *b*) and ascertainment bias toward high levels of shared polymorphism (ascertainment scheme III) (Additional file 1: Table S3). Ascertainment scheme III reflects the over-representation of within-group polymorphism observed in our empirical data. However, this ascertainment scheme still under-represents the excess of polymorphisms in European cattle observed in empirical data.

### F_ST_

*F*_*ST*_ values were calculated for each pair of populations under each scenario and are reported in Table 2. In the random sampling condition (I) pairwise *F*_*ST*_ was correlated as expected with the migration parameters in the three simulation conditions (*a*, *b*, *c*). However, ascertainment bias that inflated within-Europe polymorphism (II) decreased apparent differentiation between the European and Indian populations. In the no-migration scenario (*a*, II) the effect of this bias was sufficient to decrease European-Indian *F*_*ST*_ below that observed in the high migration scenario with or without ascertainment bias (*c*). In the ascertainment scheme biased toward increased polymorphism across all groups (III), pairwise *F*_*ST*_ values were consistently lower than in the unbiased treatment. Two-way ANOVA found highly significant effects of ascertainment scheme, demographic scenario, and the interaction between them for all three pairwise *F*_*ST*_ measures (Europe–Africa, Europe–India, Africa–India; Additional file 1: Table S4).

**Table 2:** **Mean multilocus** ***F***_***ST***_ **values (± standard deviation) calculated for each pair of populations**

	**I**			**II**			**III**		
**a**		Eur	Afr		Eur	Afr		Eur	Afr
	Afr	0.16 ± 0.01		Afr	0.15 ± 0.01		Afr	0.13 ± 0.00	
	Ind	0.79 ± 0.01	0.79 ± 0.01	Ind	0.49 ± 0.01	0.65 ± 0.01	Ind	0.55 ± 0.01	0.55 ± 0.01
		Eur	Afr		Eur	Afr		Eur	Afr
***b***	Afr	0.15 ± 0.01		Afr	0.15 ± 0.00		Afr	0.14 ± 0.01	
	Ind	0.66 ± 0.01	0.64 ± 0.01	Ind	0.58 ± 0.01	0.68 ± 0.01	Ind	0.57 ± 0.01	0.54 ± 0.01
		Eur	Afr		Eur	Afr		Eur	Afr
***c***	Afr	0.22 ± 0.02		Afr	0.16 ± 0.01		Afr	0.17 ± 0.01	
	Ind	0.68 ± 0.01	0.39 ± 0.01	Ind	0.57 ± 0.00	0.44 ± 0.01	Ind	0.56 ± 0.01	0.32 ± 0.01

(*a*) No migration; (*b*) low taurine–indicine gene flow since domestication; and (*c*) low taurine–indicine gene flow since domestication, combined with higher recent indicine to Africa gene flow. Ascertainment schemes: (I) random; (II) biased towards polymorphism in Europe; and (III) biased towards polymorphism in multiple lineages.

Calculated using Genepop [51].

### Principal components analysis

**Figure 4:**
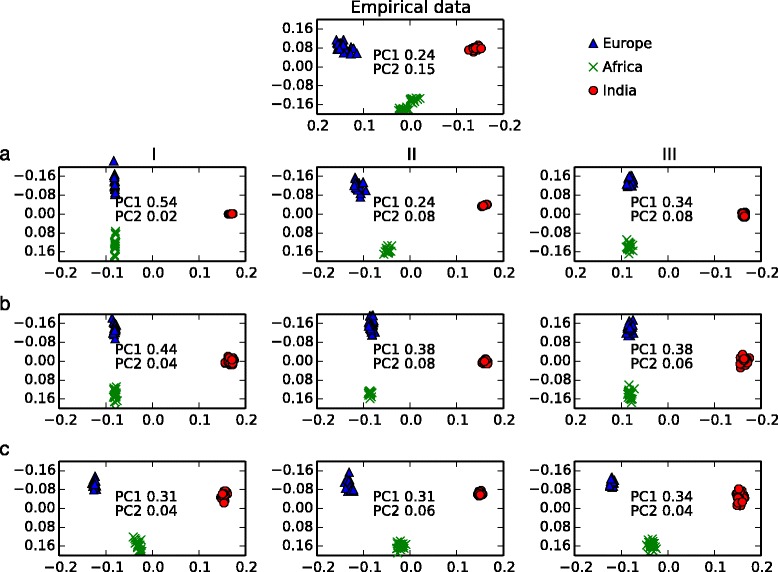
**Principal components analysis performed on 1,000-marker subsets of simulated data under three simulated migration schemes and three simulated ascertainment-bias conditions, as compared to the empirical data. (** ***a*** **)** No migration; **(** ***b*** **)** low taurine–indicine gene flow; and **(** ***c*** **)** low taurine–indicine gene flow, plus higher recent indicine to Africa gene flow. Ascertainment schemes: (I) SNPs were based on random samples of loci (no bias); (II) sampled loci were selected from those that were polymorphic within Europe; and (III) sampled loci were selected from loci that were polymorphic in two or more subpopulations. Proportions of variation accounted for by the first two PC axes are labeled on the figure.

Principal component projections of the data under each migration scenario (*a*, *b*, and *c* as described above) and ascertainment scheme (I, II, and III as described above) are shown in Figure 4. The proportion of variation accounted for by the first two principal component axes are reported in Figure 4 and with standard deviations in Additional file 1: Table S1. In all principal components analyses, the major axis of variation (PC1) differentiated taurine and indicine genotypes, and the second axis of variation (PC2) differentiated European and African taurine cattle. The proportion of variation captured by PC1, which represents the taurine–indicine split, decreased with increased gene flow in the unbiased ascertainment treatments, whereas this relationship was removed or reversed in the biased treatments (Additional file 1: Table S5). In addition, differences in ascertainment scheme significantly affect the relative PC1 score of admixed African lineages, under migration treatments *a* and *c*, as analyzed by ANOVA: (*a*) *F* = 5921, *P* = <0.0001; (*b*) *F* = 2.38, *P* = 0.09; and (*c*) *F* = 78.14, *P* = <0.0001. The strongest impact of ascertainment bias on the relative PC1 score of African individuals was in the no-migration scenario (*a*). In this scenario, under ascertainment schemes I and III, the correct inference of no admixture was inferred from the simulated data. However, in the treatment biased toward European polymorphism (II), 23% indicine ancestry was inferred in African cattle (Additional file 1: Table S6). Under the highest migration scenario (*c*), ascertainment scheme II also had the strongest impact on inferred admixture (41%), compared to only 31–32% inferred admixture under ascertainment schemes I and III to 41% under ascertainment scheme II.

The lowest residual sum of squares following Procrustes superposition between the empirical data and simulated data was under the moderate migration (*b*) and European-polymorphism biased (II) treatment (Additional file 1: Table S7). Therefore, the overall distance between the PCA locations of individuals in the empirical data and those simulated in this treatment was lowest. In all cases, coordinates were significantly more similar across treatments than would be expected by chance (*P* < 0.0001, based on a randomization test).

## Discussion

### Effects of subpopulation ascertainment bias

We found that subpopulation bias in the selection of SNP loci can affect inferences of population history. The type of ascertainment bias affects both the direction and extent of deviation in estimates of both *F*_*ST*_ and the population structure revealed by PCA.

As described in Albrechtsen *et al.* [20], selection of loci that are polymorphic within populations decreases the estimates of *F*_*ST*_ between populations. This decrease in measured *F*_*ST*_ suggests lower differentiation between populations than would be estimated from unbiased data. However, subpopulation-biased ascertainment can inflate *F*_*ST*_ as well [20]. Multiple studies have shown inflated *F*_*ST*_ values calculated from ascertained SNPs compared to whole genome sequence data [20,59]. Across our simulated data sets, we found that *F*_*ST*_ values decreased when biases inflated polymorphism in at least one of the compared populations. More problematically, at high biases toward shared polymorphism (III), *F*_*ST*_ values varied little across gene flow regimes. These results suggest that ascertainment bias may obscure information about actual population differentiation as estimated by *F*_*ST*_ values in empirical SNP data, and limit the ability of researchers to differentiate among demographic scenarios. In addition, *F*_*ST*_ values can depend heavily on the level of variation present in a sample, and the frequency of the most frequent allele [60]. Indeed, Jost [61] argued that *F*_*ST*_ was so affected by genetic diversity that it should not be used as a measure of population differentiation, gene flow, or relatedness. Based on our simulation results we do not recommend using *F*_*ST*_ to estimate demographic relationships using SNP data.

The effects of ascertainment bias on PCA are more complex. The genealogical interpretation of PCA on SNP data usually assumes that the first principal component (PC) axis captures the deepest coalescent split in the tree, and subsequent axes capture later splits [37]. In all simulated cases this interpretation was correct. However, that relationship should not be challenging to reconstruct. Admixed populations should fall between their two ancestral populations, and the proportion of ancestry inherited from each can be estimated linearly [37]. This interpretation assumes that SNP ascertainment will have a simple and predictable effect on PC projections with little influence on the relative placing of samples, except in the most extreme cases. However, in our analysis, the ascertainment scheme did impact the relative placing of simulated samples in some cases. In particular, the position of the African samples with respect to the PC1 axis was affected by an ascertainment scheme that favored selection of European polymorphisms in demographic scenario (*a*) (Figure 4). The change in relative PC1 score can be important for population genetic inference, because differences in the PC1 coordinates of the African samples can be interpreted as the difference in their proportion of admixed ancestry [10,37]. In migration scenarios *a* and *c*, selection for polymorphism in Europe (II) significantly overestimated indicine ancestry of African cattle in comparison to using randomly selected SNPs (I) (Additional file 1: Table S6). Our Procrustes superposition analyses suggest that this overestimation is due to rotation of the PC axes rather than absolute deviation in the relative centroid distances. These results show that care must be taken in interpreting PCA analyses of SNP data that are biased toward polymorphisms found in only one population.

Although variation in ascertainment bias interacted with migration to affect inference of migration based on PC1, this was not reflected in the Procrustes residual sums of squares. The Procrustes metric measures the overall deviations in the relative locations in the two-dimensional PCA coordinate space of the samples. The Procrustes results reflect that differences between ascertainment scheme affect rotation of the points relative to the axes, rather than relative to the other sampled individuals. Therefore, although ascertainment bias can affect the interpretation of PC1 as the deepest coalescent split (as described in [37]), inference of relationships among populations is less affected by population-based ascertainment bias, and is robust to biases that favor the sampling of polymorphic sites.

Recent analyses of human SNP data have made an effort to select polymorphisms within the population of interest (e.g., [62]), but subpopulation ascertainment bias is likely to continue to be a concern as panels of variable SNP loci are developed in other species [12]. Our empirical SNP chip data was generated for domesticated cattle, a group for which species relationships are not defined consistently. Some authors treat the taurine and indicine lineages as distinct species (*Bos taurus* and *Bos indicus*), whereas others treat them as subspecies (*Bos taurus taurus* and *Bos taurus indicus*). Irrespective of the naming conventions, domesticated cattle as a group capture a deep divergence between populations, and is therefore useful for examining the properties of SNP ascertainment bias across wider divergence times than those found in many model organisms. Subsets of SNPs that are informative about population structure within subpopulations may not be informative when applied to larger geographic samples [63]. The effects of bias may be even stronger when SNP panels are applied across even more divergent species, because fewer polymorphisms will be shared among these lineages as differences become fixed through time. Under these conditions, estimates of diversity in lineages closely related to the ascertainment group will be artificially inflated compared to lineages that are distantly related to the ascertainment group. Furthermore, SNPs that have been selected to differentiate between two species may result in misleading inferences about relationships among populations within other species.

As costs of sequencing continue to decrease, it is becoming more feasible to generate whole-genome sequence data, even from non-model organisms. Such data do decrease the effects of ascertainment bias on inference relative to SNP samples [59]. Nonetheless, even in whole genome sequence data, alignment to a divergent reference genome [64] or removing sites with a high proportion of missing data across taxa can generate ascertainment bias in the analyzed data set [65].

### Application to inference of cattle population history

Murray *et al.* [38] estimated the demographic parameters that we used in our simulations, using 37 kb of autosomal DNA sequenced in cattle from Europe, Africa, and the Indian subcontinent. Although these loci were selected based on their variability, this data set lacks the strong ascertainment bias of the SNP data set. The SNP panel captures many sites that are polymorphic in both taurine and indicine cattle. Figure 3 demonstrates that if our demographic simulations are accurate, the 50 K bovine SNP panel data greatly over-represents both European and African polymorphism and shared polymorphism among groups. This SNP panel also underestimates indicine diversity.

Based on inferences from ascertained SNP data, there are remarkably high levels of shared polymorphisms maintained between indicine and taurine lineages across 280 kya of divergence. This prevalence of deep coalescence events is particularly surprising given the estimates from mtDNA of extremely narrow bottlenecks associated with domestication [66]. MacEahern *et al.* [67] found that approximately 10% of all ascertained 50 K SNP chip polymorphisms that segregate in two taurine breeds (Angus and Holstein) also segregate in at least one of Bison, Yak, or Banteng. Matukumalli *et al.* [45] also found that 1–5% of SNPs in the 50 K panel were polymorphic in other *Bos* species, and some were variable in multiple outgroup species. Taken together, these results suggest that this SNP panel is capturing sites with unusual evolutionary histories, such as older polymorphisms that have been maintained through selection [59]. Nonetheless, even in autosomal data, shared polymorphisms between taurine and indicine lineages are numerous enough that the best-fit model requires significant gene flow between the lineages, strong balancing selection on segregating sites, very large population sizes, or some combination of these factors [38,68].

By comparing the simulation results with the estimates based on empirical data from cattle, we can assess the effects of different types of ascertainment bias on estimates of population history. Biases toward shared polymorphisms (Table 2: II, III) decreased estimates of *F*_*ST*_ by increasing the contribution of shared among-group variation. Our simulated data consistently had lower within-taurine African–European divergence than in observed data. Biased samples in the highest gene flow regime (Table 2: IIc, IIIc) did reflect the observed divergence between African and indicine populations. This result suggests that indicine gene flow into Africa likely occurred at a higher rate than estimated by Murray *et al.* [38], although these authors did not explicitly address African taurine cattle.

There are many alternative combinations of demographic processes and ascertainment biases that could produce the patterns we observed in empirical data, and we do not compare among all possibilities. In addition, all simulation conditions reflected less divergence between European and African cattle, than were observed in our empirical data, consistent with the reduced *F*_*ST*_ values. This suggests that these lineages may have diverged more than 15 kya.

There are several potentially important demographic factors that were not addressed in our simulations or Murray *et al.*’s [38] demographic analyses. In both cases, major continental groups were treated as panmictic populations, which is biologically unlikely. Population substructuring within each of these regions could affect inference of demographic parameters in several ways. Within-population structure can bias estimates of population sizes, often resulting in apparent recent population size declines [69-71]. These effects of population structuring can also interact with gene flow and the sampling scheme to cause spurious inference of bottlenecks [72,73]. Although the empirical data used here do include extensive within-population sampling, which should mitigate some of the potential issues caused by overdispersed sampling schemes, overdispersed sampling nonetheless likely affected both our inferences and the demographic model of Murray *et al.* [38]. New whole-genome approaches for estimating the history of recent population size may contribute better estimates for these parameters in the near future [74,75].

## Conclusions

The sample size of ascertainment sets strongly affects the limit of the minor allele frequency that can be captured in a SNP panel. Although we did not directly explore the effects of different sample sizes of subpopulations in our analyses, our ascertainment bias schemes capture the effects of uneven sampling across populations. Biasing selection of sites to those that are polymorphic within a single population is analogous to having larger sample sizes for that subpopulation. In either case, more sites that are polymorphic in targeted population are included in later analyses.

Although issues of ascertainment bias have been addressed extensively in human data, studies of non-model organisms often involve deeper divergences among sampled populations. Our simulation results demonstrate the importance of taking ascertainment bias into account when using SNP data for phylogeographic analysis. Despite the limitations of SNP studies, the strongest signal in our example empirical and simulated data sets for cattle—the differentiation between indicine and taurine cattle —was consistent across treatments, and was robust to even strong ascertainment bias. Bias toward polymorphisms found in only a single population affects inferences of population relationships more strongly than does bias toward interpopulational polymorphisms.

### Availability of supporting data

#### Data deposition

The empirical and simulated data, as well as the python code used for simulation and analyses, have been deposited in the Dryad repository (datadrayd.org; *doi:10.5061/dryad.ht0hs upon publication*).

## Additional file

Additional file 1: Table S1. Commands used for the simulations in this study. **Table S2.** Counts of polymorphic sites within, and shared among, geographic regions for the simulation replicate shown in Figure 3. **Table S3.** Mean deviation from empirical data of simulated polymorphism counts. **Table S4.** Two-way ANOVA on *F*_*ST*_ values. **Table S5.** Mean proportion of variation captured by PC1 and PC2. **Table S6.** Estimated proportion of admixture in the African cattle lineage. **Table S7.** Residual sums of squares across five replicates of Procrustes analyses.

## Abbreviations

SNP: Single nucleotide polymorphism, used here in the broad sense to mean variable site
PCA: Principal components analysis
ANOVA: Analysis of variance
