# Supplementary material for: How do SNP ascertainment schemes and population demographics affect inferences about population history?
Source: BMC Genomics. 2015 Apr 3;16(1):266. doi: 10.1186/s12864-015-1469-5 (PMC4428227; doi:10.1186/s12864-015-1469-5)
Supplement: Additional file 1: Table S1. — Commands used for the simulations in this study. Table S2. Counts of polymorphic sites within, and shared among, geographic regions for the simulation replicate shown in Figure 3. Table S3. Mean deviation from empirical data of simulated polymorphism counts. Table S4. Two-way ANOVA on F ST values. Table S5. Mean proportion of variation captured by PC1 and PC2. Table S6. Estimated proportion of admixture in the African cattle lineage. Table S7. Residual sums of squares across five replicates of Procrustes analyses. [file 12864_2015_1469_MOESM1_ESM.docx]

**Table S1.** Commands used for simulations in this study

| ***a*** | ms 150 100000 -t 0.180000 -I 3 50.000000 50.000000 50.000000 0 -en 0 1 0.5 -en 0 2 0.5 -en 0 3 1 -ej 0.050000 1 2 -ej 0.933333 3 2 -en 0.120000 2 0.010000 -en 0.133333 2 1.000000 |
| --- | --- |
| ***b*** | ms 150 100000 -t 0.180000 -I 3 50.000000 50.000000 50.000000 0 -en 0 1 0.5 -en 0 2 0.5 -en 0 3 1 -ej 0.050000 1 2 -ej 0.933333 3 2 -en 0.120000 2 0.010000 -en 0.133333 2 1.000000 -em 0.05 2 3 0.87 -em 0.05 3 2 0.05 |
| ***c*** | ms 150 100000 -t 0.180000 -I 3 50.000000 50.000000 50.000000 0 -en 0 1 0.5 -en 0 2 0.5 -en 0 3 1 -ej 0.050000 1 2 -ej 0.933333 3 2 -en 0.120000 2 0.010000 -en 0.133333 2 1.000000 -em 0 2 3 8 -em 0.05 2 3 0.87 -em 0.05 3 2 0.05 |
|  |  |

**Table S2.** Counts of polymorphic sites within, and shared among, geographic regions for the simulation replicate shown in Figure 3.

| **Data set** | **Sampling**  **scheme** | **Geographic regions** | | | | | | |
| --- | --- | --- | --- | --- | --- | --- | --- | --- |
|  |  | **Europe** | **Africa** | **India** | **Europe and Africa** | **Europe**  **and**  **India** | **Africa and**  **India** | **Europe, Africa, and**  **India** |
| **Empirical data** | **All** | 6325 | 1452 | 1373 | 9540 | 2873 | 2463 | 17046 |
|  | **Random** | 139 | 25 | 27 | 203 | 56 | 42 | 377 |
| ***a*** | **All** | 3848 | 3991 | 18573 | 1394 | 1 | 3 | 12 |
|  | **I** | 84 | 90 | 364 | 26 | 0 | 0 | 0 |
|  | **II** | 585 | 13 | 77 | 236 | 0 | 0 | 2 |
|  | **III** | 16 | 15 | 83 | 775 | 0 | 1 | 28 |
| ***b*** | **All** | 4958 | 4964 | 17840 | 2592 | 691 | 695 | 871 |
|  | **I** | 102 | 96 | 394 | 61 | 21 | 20 | 23 |
|  | **II** | 449 | 26 | 83 | 244 | 60 | 2 | 84 |
|  | **III** | 18 | 23 | 77 | 272 | 81 | 77 | 391 |
| ***c*** | **All** | 4272 | 9294 | 9278 | 3240 | 142 | 9070 | 1339 |
|  | **I** | 98 | 200 | 193 | 62 | 4 | 185 | 38 |
|  | **II** | 395 | 46 | 42 | 307 | 10 | 28 | 130 |
|  | **III** | 17 | 39 | 34 | 152 | 10 | 439 | 25 |

**Table S3.** Mean deviation from empirical data of simulated polymorphism counts ± standard deviation across five replicates, presented within, and shared among, geographic regions. The summed absolute values of deviations across all seven categories are also presented. Results are presented for nine simulation conditions (three migration scenarios: *a*, *b*, and *c*; and three ascertainment schemes: I, II, and III; see text for details). The smallest sum of absolute deviations (combination *b*-III) is shown in bold.

| **Data Set** | **Sampling scheme** | **Summed total deviations** | **Geographic region** | | | | | | |
| --- | --- | --- | --- | --- | --- | --- | --- | --- | --- |
|  |  |  | **Europe** | **Africa** | **India** | **Europe**  **and Africa** | **Europe**  **and India** | **Africa**  **and India** | **Europe, Africa,**  **and India** |
| ***a*** | **I** | 1.46±0.21 | 0.01±0.01 | 0.11±0.01 | 0.61±0.01 | 0.17±0.02 | 0.07±0.00 | 0.05±0.00 | 0.43±0.01 |
|  | **II** | 1.15±0.19 | 0.50±0.01 | 0.02±0.00 | 0.05±0.00 | 0.02±0.01 | 0.07±0.00 | 0.05±0.00 | 0.43±0.01 |
|  | **III** | 1.34±0.21 | 0.13±0.01 | 0.02±0.00 | 0.05±0.01 | 0.62±0.02 | 0.07±0.00 | 0.05±0.01 | 0.40±0.02 |
| ***b*** | **I** | 1.26±0.18 | 0.01±0.01 | 0.11±0.01 | 0.51±0.02 | 0.15±0.02 | 0.05±0.01 | 0.03±0.01 | 0.40±0.01 |
|  | **II** | 0.84±0.14 | 0.34±0.03 | 0.01±0.01 | 0.05±0.01 | 0.04±0.03 | 0.01±0.01 | 0.05±0.01 | 0.35±0.01 |
|  | **III** | **0.34±0.04** | 0.13±0.01 | 0.01±0.01 | 0.05±0.01 | 0.08±0.04 | 0.02±0.01 | 0.02±0.01 | 0.03±0.03 |
| ***c*** | **I** | 1.23±0.11 | 0.03±0.01 | 0.22±0.02 | 0.22±0.01 | 0.13±0.02 | 0.07±0.01 | 0.17±0.02 | 0.39±0.02 |
|  | **II** | 0.75±0.11 | 0.26±0.01 | 0.01±0.01 | 0.01±0.01 | 0.09±0.02 | 0.05±0.00 | 0.02±0.01 | 0.30±0.01 |
|  | **III** | 0.85±0.13 | 0.13±0.01 | 0.01±0.00 | 0.00±0.00 | 0.06±0.02 | 0.06±0.00 | 0.41±0.01 | 0.17±0.02 |

**Table S4.** Two-way ANOVA on *F_ST_* values by ascertainment scheme and demographic scenario for each pairwise comparison between populations.

| **Pairwise comparison** | **Factor** | **Degrees of freedom** | **Sum of Squares** | **Mean square** | **F** | **P - value** |
| --- | --- | --- | --- | --- | --- | --- |
| **Europe-Africa** | Ascertainment scheme | 2 | 0.0277 | 0.0139 | 129.86 | P<<0.0001 |
|  | Demographic scenario | 3 | 0.0519 | 0.0173 | 162.06 | P<<0.0001 |
|  | Interaction | 6 | 0.0052 | 0.0009 | 8.11 | P<<0.0001 |
|  | Residual | 40 | 0.0043 | 0.0001 | - | - |
| **Europe-India** | Ascertainment scheme | 2 | 0.0796 | 0.0398 | 327.65 | P<<0.0001 |
|  | Demographic scenario | 3 | 0.3251 | 0.1084 | 892.47 | P<<0.0001 |
|  | Interaction | 6 | 0.0735 | 0.0123 | 100.89 | P<<0.0001 |
|  | Residual | 40 | 0.0049 | 0.0001 | - | - |
| **India-Africa** | Ascertainment scheme | 2 | 0.1162 | 0.0581 | 448.53 | P<<0.0001 |
|  | Demographic scenario | 3 | 0.8615 | 0.2872 | 2216.26 | P<<0.0001 |
|  | Interaction | 6 | 0.0636 | 0.0106 | 81.75 | P<<0.0001 |
|  | Residual | 40 | 0.0052 | 0.0001 | - | - |

**Table S5.** Mean proportion of variation captured by PC1 and PC2 (± standard deviations). Values were calculated for 1,000 SNP data subsets for both empirical data and under each of nine combinations of simulation conditions (three migration scenarios: *a*, *b*, and *c*; and three ascertainment schemes: I, II, and III; see text for details)

| Empirical data: PC1: 0.24 ± 0.01; PC2: 0.15 ± 0.00 | | | | | | | |
| --- | --- | --- | --- | --- | --- | --- | --- |
| Simulated data: | | | | | | | |
|  | | | **I** | | **II** | | **III** |
| ***a*** | PC1 | 0.52±0.01 | | 0.23±0.01 | | 0.33±0.01 | |
|  | PC2 | 0.02±0.00 | | 0.08±0.00 | | 0.08±0.00 | |
| ***b*** | PC1 | 0.42±0.01 | | 0.37±0.01 | | 0.38±0.01 | |
|  | PC2 | 0.03±0.00 | | 0.08±0.00 | | 0.06±0.00 | |
| ***c*** | PC1 | 0.32±0.01 | | 0.31±0.00 | | 0.36±0.01 | |
|  | PC2 | 0.04±0.00 | | 0.06±0.00 | | 0.05±0.00 | |

**Table S6.** Estimated proportion of admixture (± standard deviations) in the African cattle lineage, as inferred based on the relative position on PC1 of African cattle, as described in McVean (2009). Negative values are not significantly different from 0, and imply no admixture. Estimates are presented for the empirical data samples as well as nine combinations of simulation conditions (three migration scenarios: *a*, *b*, and *c*; and three ascertainment schemes: I, II, and III; see text for details)

| Empirical data: 0.3948 ± 0.111 | | | |
| --- | --- | --- | --- |
| Simulated data: | | | |
|  | **I** | **II** | **III** |
| ***a*** | 0.0008±0.004 | 0.2332±0.020 | -0.0147±0.028 |
| ***b*** | -0.0021±0.010 | 0.0204±0.179 | -0.0066±0.021 |
| ***c*** | 0.3256±0.024 | 0.4100±0.110 | 0.3143±0.020 |

**Table S7.** Residual sums of squares (± standard deviation) across five replicates of Procrustes analyses comparing principal component analyses of empirical data to simulated data. Residual sums of squares are presented for nine combinations of simulation conditions (three migration scenarios: *a*, *b*, and *c*; and three ascertainment schemes: I, II, and III; see text for details)

|  | **I** | **II** | **III** |
| --- | --- | --- | --- |
| ***a*** | 0.0379 ± 0.004 | 0.0224 ± 0.003 | 0.0189 ± 0.001 |
| ***b*** | 0.0298 ± 0.004 | 0.0184 ± 0.002 | 0.0214 ± 0.002 |
| ***c*** | 0.0251 ± 0.004 | 0.0221 ± 0.001 | 0.0248 ± 0.004 |
